# Mesothelin-Binding Peptide Inhibits Cell Migration and Enables Targeted Delivery of a Mitochondrial-Membrane-Damaging Peptide to Pancreatic Tumors

**DOI:** 10.34133/bmr.0361

**Published:** 2026-05-11

**Authors:** Min-Sung Park, Gowri Rangaswamy Gunassekaran, Seok-Min Lee, Sri Murugan Poongkavithai Vadevoo, Dong-Gyun Jo, Soyoun Kim, Eunji Ha, Sang-Kyun Kim, Hyejin Ahn, Sang-Jeon Chung, Gyeongmin Kang, Mi Rim Lee, Sang Myung Woo, Yun-Hee Kim, Byungheon Lee

**Affiliations:** ^1^ Department of Biochemistry and Cell Biology, School of Medicine, Kyungpook National University, Daegu 41944, Republic of Korea.; ^2^ BK21 Plus KNU Biomedical Convergence Program, Department of Biomedical Science, School of Medicine, Kyungpook National University, Daegu 41944, Republic of Korea.; ^3^ CMRI, School of Medicine, Kyungpook National University, Daegu 41944, Republic of Korea.; ^4^ Department of Molecular Medicine, School of Medicine, Kyungpook National University, Daegu 41944, Republic of Korea.; ^5^ Laboratory Animal Center, K-Medi Hub, Daegu 41061, Republic of Korea.; ^6^ College of Pharmacy, Sungkyunkwan University, Suwon 16419, Republic of Korea.; ^7^ Research Institute and Hospital, National Cancer Center, Goyang 10408, Republic of Korea.; ^8^ Graduate School of Cancer Science and Policy, National Cancer Center, Goyang 10408, Republic of Korea.

## Abstract

Mesothelin (MSLN) is up-regulated in many tumors, including pancreatic tumors, making it a promising therapeutic target. We used a phage-displayed peptide library to identify an MSLN-binding peptide. CTILWSLTC, synthesized in cyclic form (MSLNpep), was selected from the library. MSLNpep bound preferentially to MSLN-high versus MSLN-low cells, internalized efficiently, and bound recombinant MSLN more strongly than a control peptide. MSLNpep treatment reduced migration and invasion in MSLN-high tumor cells. To enable targeted cytotoxicity, we generated chimeras linking MSLNpep to L-form or D-form mitochondrial-membrane-damaging peptides (MSLNpep-KLA and MSLNpep-kla, respectively). MSLNpep-kla—and, to a lesser extent, MSLNpep-KLA—exhibited selective cytotoxicity toward MSLN-high versus MSLN-low cells. Systemic MSLNpep-kla, but not MSLNpep-KLA, suppressed orthotopic pancreatic tumor growth, increased survival, and produced no detectable hepatic or renal toxicity in mice. MSLNpep-kla also induced cytotoxicity in patient-derived pancreatic cancer organoids proportionate to MSLN expression. Analysis of a public single-cell RNA-sequencing dataset of human pancreatic cancer tissues revealed that *MSLN* was predominantly expressed in malignant ductal cells while showing negligible expression in normal ductal cells and stromal cells. These findings demonstrate that MSLNpep inhibits tumor-cell migration and enables targeted delivery of therapeutic payloads to MSLN-high pancreatic tumors, supporting its use as a peptide ligand for cancer therapy.

## Introduction

Pancreatic cancer is a leading cause of cancer-related death worldwide, with a 5-year survival of approximately 10%, largely because diagnosis often occurs after extensive local invasion and distant metastasis [1,2]. Standard chemotherapy (e.g., gemcitabine) remains the mainstay but is limited by frequent resistance [3,4]. Few validated targets or biomarkers exist for precision therapy, and pancreatic cancer is generally immunologically “cold”, conferring resistance to immune-checkpoint blockades [5]. Dense, fibrotic stromal collagen further impedes drug delivery [6]. Accordingly, substantial unmet needs persist in the management of pancreatic cancer.

Mesothelin (MSLN) is a cell-surface protein. An MSLN precursor protein (71 kDa) is linked to the cell membrane via a glycosylphosphatidylinositol linker and is cleaved by furin into an N-terminal-secreted form, megakaryocyte potentiating factor (31 kDa), and a C-terminal membrane-bound form, MSLN (40 kDa) [7]. In the tumor microenvironment, MSLN may be further cleaved near the cell membrane and released as soluble MSLN [8]. Soluble MSLN is typically more abundant in tumor tissue than in blood [9]. Notably, MSLN is up-regulated in approximately 80% of patients with pancreatic cancer, particularly in advanced disease, while being expressed at negligible levels in normal tissues and PanIN [10–12]. Its expression is associated with aggressive features of pancreatic cancer [13]. Additionally, MSLN has been implicated in migration and invasion of mesothelioma cells and in epithelial–mesenchymal transition in lung cancer [14,15]. These observations nominate MSLN as a target and biomarker of pancreatic cancer.

To date, antibody-based therapeutics have been investigated as MSLN-targeted therapies, including MSLN-targeted immunotoxins [8], antibody–drug conjugates [16,17], and chimeric antigen receptor T (CAR-T) cells expressing anti-MSLN antibodies [18,19]. Compared with antibodies, peptides typically show lower affinity, faster renal clearance, and greater susceptibility to peptidases; in contrast, peptides offer superior tissue penetration, efficient cellular internalization, and reduced immunogenicity [20–22]. These advantages are particularly relevant to pancreatic tumors with dense, fibrotic microenvironments. We have used phage-displayed peptide libraries to identify ligands to cancer targets such as interleukin-4 receptor and tumor necrosis factor superfamily 19-like and peptides that bind to and inhibit the programmed death-ligand 1 immune checkpoint [23–25]. Here, we sought to discover a peptide that selectively binds MSLN and to exploit it for targeted delivery of therapeutics to MSLN-high pancreatic tumors.

## Materials and Methods

### Cell culture

HEK 293T (human embryonic kidney) cells and AsPC-1, MIA PaCa-2, PANC-1, and BxPC-3 (human pancreatic cancer) cells were obtained from the American Type Culture Collection (Manassas, VA). KPC (mouse pancreatic cancer) cells were provided by Dr. Kazuki Sugahara (Columbia University, USA). HEK 293T, PANC-1, and MIA PaCa-2 cells were maintained in Dulbecco’s modified Eagle’s medium (DMEM). AsPC-1 and BxPC-3 cells were maintained in RPMI-1640. KPC cells were maintained in DMEM-high glucose. Media were supplemented with 10% fetal bovine serum (FBS; HyClone, Logan, UT), 100 U/ml penicillin, and 100 μg/ml streptomycin. Cells were cultured in a humidified incubator at 37 °C with 5% CO_2_.

### Preparation of MSLN-overexpressing cells

The pCMV6-AC-GFP mammalian expression vector encoding green fluorescent protein (GFP)-tagged MSLN was purchased from Origene Technologies Inc. (Rockville, MD). HEK 293T cells were seeded in 6-well plates at 2 × 10^5^ cells/well and incubated for 24 h to reach 70% to 80% confluence. Lipofectamine 3000 (10 μl; Thermo Fisher Scientific, Waltham, MA) was diluted in 250 μl Opti-MEM (Thermo Fisher Scientific), combined with 4 μg MSLN expression vector, and incubated for 30 min at room temperature (RT). Cells were then incubated with the transfection mixture for 4 to 6 h. The mixture was replaced with culture medium containing 10% FBS, and cells were incubated for an additional 48 h. MSLN expression after transfection was assessed by immunofluorescence using an anti–human MSLN antibody (Thermo Fisher Scientific) and confocal microscopy (Nanoscope Systems, Daejeon, Korea).

### Biopanning for MSLN-binding peptides

We used a CX7C phage-displayed peptide library containing cysteines at the N and C termini and 7 randomized amino acids; hydrophobic residues occurred at least once per heptamer. To remove phages that bound nonspecifically to HEK 293T cells, 1 × 10^9^ plaque-forming units of the library were incubated with nontransfected HEK 293T cells at 4 °C for 1 h. The supernatant containing unbound phages was collected and incubated with HEK 293T cells transfected with the MSLN expression vector at 4 °C for 1 h for selection. Cell-bound phages were eluted using BL21 host cells at RT for 10 min and amplified for the next round of biopanning.

### Phage-cell-binding ELISA

MSLN-transfected HEK 293T cells (1 × 10^4^ per well) in 96-well plates were blocked with 5 mg/ml bovine serum albumin (BSA) at RT for 1 h. After washing 3 times with Tris-buffered saline containing 0.1% Tween-20, 100 μl of individual phage clones (1 × 10^7^ plaque-forming units per well) were added and incubated at 4 °C for 1 h. After washing, horseradish-peroxidase-conjugated anti-T7 tail fiber antibody (Merck, Darmstadt, Germany) diluted 1:10,000 in blocking buffer was added and incubated at RT for 1 h. After washing, 3,3′,5,5′-tetramethylbenzidine substrate (Pierce, Rockland, IL) was added and incubated at RT for 10 to 15 min. The reaction was stopped by adding 100 μl of 2 M H_2_SO_4_, and absorbance was measured at 450 nm using a microplate reader (Thermo Fisher Scientific).

### Peptide synthesis

Phage DNA regions flanking the inserted peptide sequences were amplified by polymerase chain reaction and sequenced by Macrogen Inc. (Seoul, Korea). Peptide sequences were analyzed using ClustalW. Disulfide-cyclized CTILWSLTC (molecular weight: 1,037.6) was synthesized and purified by high-performance liquid chromatography to >90% purity, and mass was confirmed by matrix-assisted laser desorption/ionization–time of flight mass spectrometry (Anygen Inc., Gwangju, Korea). Peptides were conjugated at the N terminus with fluorescein isothiocyanate (FITC), tetramethylrhodamine-5-maleimide (TAMRA), or biotin. Lyophilized peptides were reconstituted in distilled water to 10-mM stock solutions and diluted with dimethyl sulfoxide to working concentrations. The NSSSVDK peptide, present in the phage coat protein, served as the control.

### Immunofluorescence microscopy

Cells (1 × 10^5^ per well) were seeded onto 4-well chamber slides, incubated overnight, and blocked with 1% BSA at RT for 1 h to reduce nonspecific binding. To assess peptide binding, cells were incubated with 25 μM FITC (green)-conjugated peptides at 4 °C for 1 h. For cells expressing GFP (green), TAMRA (red)-conjugated peptides were used instead. After incubation, cells were fixed in 4% paraformaldehyde and incubated with an anti–human polyclonal MSLN antibody (1:200; Thermo Fisher Scientific). To examine internalization, cells were incubated with 25 μM FITC-conjugated peptides at 37 °C for 2 h. For competition assays, cells were preincubated with anti–human polyclonal MSLN antibody (Thermo Fisher Scientific) at RT for 1 h, then incubated with 25 μM FITC-conjugated peptides at 4 °C for 1 h. In parallel, peptide was preincubated with soluble or mature MSLN at 4 °C for 30 min and then applied to cells for an additional 30 min at 4 °C. After incubation, cells were fixed in 4% paraformaldehyde, stained with diamidino-2-phenylindole (Sigma-Aldrich, St. Louis, MO) to label nuclei, mounted with ProLong Antifade reagent (Thermo Fisher Scientific), and imaged using a confocal microscope (Nanoscope Systems).

### MSLN gene silencing and flow cytometry

Cells (3 × 10^5^ per well) cultured in 6-well plates overnight were transfected with 100 or 200 nM small interfering RNA (siRNA) against MSLN (Bioneer, Daejeon, Korea) using Lipofectamine RNAiMAX (Thermo Fisher Scientific) for 24 to 48 h. Cells were blocked in culture medium containing 1% BSA at RT for 1 h, incubated with 25 μM FITC-conjugated peptides at 4 °C for 1 h, and analyzed by flow cytometry (Thermo Fisher Scientific). Percent MSLN expression and peptide binding were quantified and analyzed using Kaluza software (Beckman Coulter, Brea, CA).

### Pull-down assays

Biotin-labeled peptides were incubated with streptavidin-coated magnetic beads (Bioclone, San Diego, CA) at RT for 1 h with gentle rotation to generate peptide–bead complexes. Cells were lysed in lysis buffer containing protease inhibitors (Thermo Fisher Scientific). Cell lysates were incubated with 50 μl peptide–bead complexes (1 μg/μl) at RT for 1 h with gentle rotation. Complexes were captured magnetically, and bound proteins were eluted with an elution buffer (Bioclone). Eluates were resolved by sodium dodecyl sulfate–polyacrylamide gel electrophoresis (SDS–PAGE), transferred to membranes, and immunoblotted with anti–human MSLN antibody (Cell Signaling Technology, Danvers, MA).

### Peptide binding by ELISA

To evaluate peptide binding to MSLN by ELISA, 96-well nickel-coated plates (Thermo Fisher Scientific) were coated at 4 °C for 24 h with 0.3 μg his-tagged soluble MSLN (residues 296 to 592) and his-tagged mature MSLN (residues 296 to 606) (provided by Professor Chang-Han Lee, College of Medicine, Seoul National University, Seoul, Korea). Wells were washed with phosphate-buffered saline containing 0.05% Tween-20. MSLN-coated wells were incubated with biotin-labeled peptides at 0, 3.125, 6.25, 12.5, 25, or 50 μM at RT for 1 h, followed by horseradish-peroxidase-conjugated streptavidin (1:10,000; Thermo Fisher Scientific) at RT for 1 h. Then, 3,3′,5,5′-tetramethylbenzidine substrate (Thermo Fisher Scientific) was added, and after 10 min, the reaction was stopped with 2 N H_2_SO_4_. Absorbance at 450 nm was measured with a microplate reader (Thermo Fisher Scientific).

### Computer-simulated structural analysis

Peptide structures were predicted using AlphaFold [26]. Docking of the peptide to MSLN (Protein Data Bank 8CX3) was performed with DiffDock [27]. DiffDock generated 100 ligand poses; model confidence scores were calculated with Neurosnap (http://neurosnap.ai).

### Cell migration and invasion assays

To assess migration, cells (2 × 10^5^ per well) were seeded into the upper chambers of Transwell-24 plates with 8-μm pores (Corning, Corning, NY). After overnight culture, upper chambers received 200 μl culture medium containing peptides at 25, 50, 100, or 200 μM, and lower chambers were filled with 600 μl culture medium containing 10% FBS. After 24 h, cells that migrated to the underside of the membranes were fixed with methanol, stained with crystal violet, and counted by light microscopy. For invasion assays, upper membranes were precoated with Matrigel (300 μg/ml; Corning).

### Immunoblot analysis

To assess MSLN expression, cell lysates (20 μg) were subjected to SDS–PAGE and immunoblotting with anti-MSLN antibody (Cell Signaling Technology). An anti-glyceraldehyde-3-phosphate dehydrogenase (GAPDH) antibody (Cell Signaling Technology) served as the loading control. To examine ERK1/2 phosphorylation, cells (5 × 10^5^ per well) in 6-well plates were serum-starved overnight and incubated with peptide (100 or 200 μM) for 2, 4, or 24 h. After incubation, cell lysates (30 μg) were analyzed by SDS–PAGE and immunoblotting with rabbit anti-phospho-ERK1/2 and rabbit anti-ERK1/2 monoclonal antibodies (Cell Signaling Technology). Horseradish-peroxidase-conjugated anti-rabbit IgG (Cell Signaling Technology) was used as the secondary antibody. Protein bands were detected using Enhanced Chemiluminescence reagents (Thermo Fisher Scientific).

### Cytotoxicity assays

Cells (5 × 10^3^ per well) in 96-well plates were incubated with peptides at various concentrations for 24 h. After treatment, cells were incubated in fresh culture medium containing 10% Cell Counting Kit-8 (Dojindo, Kumamoto, Japan) for 1 h, and absorbance at 450 nm was measured. The half-maximal inhibitory concentration was calculated using GraphPad Prism 7 (GraphPad, New York, NY).

### Antitumor therapy

Mice were purchased from Orient Bio (Seongnam, Korea) and housed in accordance with the Institutional Animal Care and Use Committee guidelines of Kyungpook National University (permission number: 2023-04-06). Mice were identified using nontoxic skin markings applied to the tail. No invasive identification procedures, such as ear notching, were used. Animals were monitored to ensure that the markings did not induce abnormal grooming, stress-related behaviors, or skin irritation. To generate an orthotopic pancreatic tumor model, AsPC-1-luc cells (2 × 10^6^) expressing luciferase were mixed 1:1 with Matrigel and inoculated into the pancreas of 6-week-old BALB/c male nude mice. Tumors were allowed to establish for 2 weeks [28]. Tumor burden was monitored by whole-body bioluminescence imaging 10 min after intraperitoneal injection of D-luciferin (Revvity, Waltham, MA; 150 mg/kg body weight) using an IVIS imaging system (PerkinElmer, Shelton, CT). Tumor-bearing mice received peptides via tail-vein injection (10 mg/kg body weight) 3 times weekly for a total of 10 doses. Mice were monitored for survival after treatment. In a separate cohort, mice were euthanized at the end of treatment, and blood was collected to measure serum liver enzymes (aspartate aminotransferase, alanine aminotransferase, and alkaline phosphatase) and kidney function markers (blood urea nitrogen and creatinine) by DGMIF (Daegu, Korea).

### Patient-derived organoid cultures

Human pancreatic cancer patient-derived organoids (PDOs) were established as described [29,30]. Pancreatic tumor tissue or ascites from patients attending the pancreatobiliary cancer clinic at the National Cancer Center (Seoul, Korea) were dissociated by combined mechanical disruption and enzymatic digestion of extracellular matrix using MACS Dissociators (Miltenyi Biotec, Rhineland, Germany). Single-cell suspensions were embedded in Cultrex reduced growth factor basement membrane extract type 2 (R&D Systems, Minneapolis, MN) in 24-well plates. After gelling, organoid growth medium was added: advanced Dulbecco’s modified Eagle’s medium/F12 (Invitrogen, Waltham, MA) containing B-27 (Invitrogen), 1.25 mM *N*-acetylcysteine (Sigma-Aldrich), 10 mM nicotinamide (Sigma-Aldrich), 10 mM HEPES (Gibco), 1% GlutaMAX (Gibco), 50 ng/ml epidermal growth factor (PeproTech, Cranbury, NJ), 100 ng/ml fibroblast growth factor 10 (PeproTech), 50 ng/ml recombinant human R-spondin 1 (Qkine, Cambridge, UK), 100 ng/ml Wingless-related integration site surrogate–Fc fusion protein (U-Protein Express BV, Utrecht, Netherlands), 1% noggin–Fc fusion protein–conditioned medium (U-Protein Express BV), 500 nM A83-01 (Tocris, Bristol, UK), and 1% Primocin (InvivoGen). Study protocols and patient-derived sample collection were approved by the Institutional Review Board of the National Cancer Center of Korea (approval nos. NCC2019-034 and NCC2021-0232). For drug-response assays, cells were seeded at 1 × 10^3^ cells per well in 384-well plates with 10% basement membrane extract. After a 3-d incubation to allow organoid formation, organoids were treated with peptides. Cell viability was measured after 5 d using the CellTiter-Glo 3D Cell Viability Assay kit (Promega, Madison, WI). Luminescence was measured using an Infinite 200 PRO spectrophotometer (Tecan, Mannedorf, Switzerland).

### Analysis of public single-cell RNA-sequencing data

To evaluate the expression profile of *MSLN* in pancreatic cancer at the single-cell level, we utilized a publicly available single-cell RNA-sequencing (scRNA-seq) dataset (accession no. CRA001160; [31]). The processed gene expression matrix and metadata were imported into R (v4.5.0) using the Seurat package (v5.4.0). We utilized the cell-type annotations provided by the original authors to distinguish between malignant ductal cells (type 2), normal ductal cells (type 1), and other stromal/immune cell populations. Gene expression levels were normalized using the LogNormalize method. The specificity of *MSLN* expression across different cell types was visualized using violin plots. Co-expression analysis between *MSLN* and the epithelial marker *KRT19* was performed using Spearman correlation analysis within the ductal cell population.

### Statistical analysis

Statistical significance was assessed using unpaired *t* test, one-way analysis of variance (ANOVA) with Tukey’s post hoc test, or 2-way - (ANOVA) followed by Tukey’s multiple comparisons test. *P* <0.05 was considered statistically significant.

## Results

### Biopanning of a phage-displayed peptide library to identify an MSLN-binding peptide

**Fig. 1.**
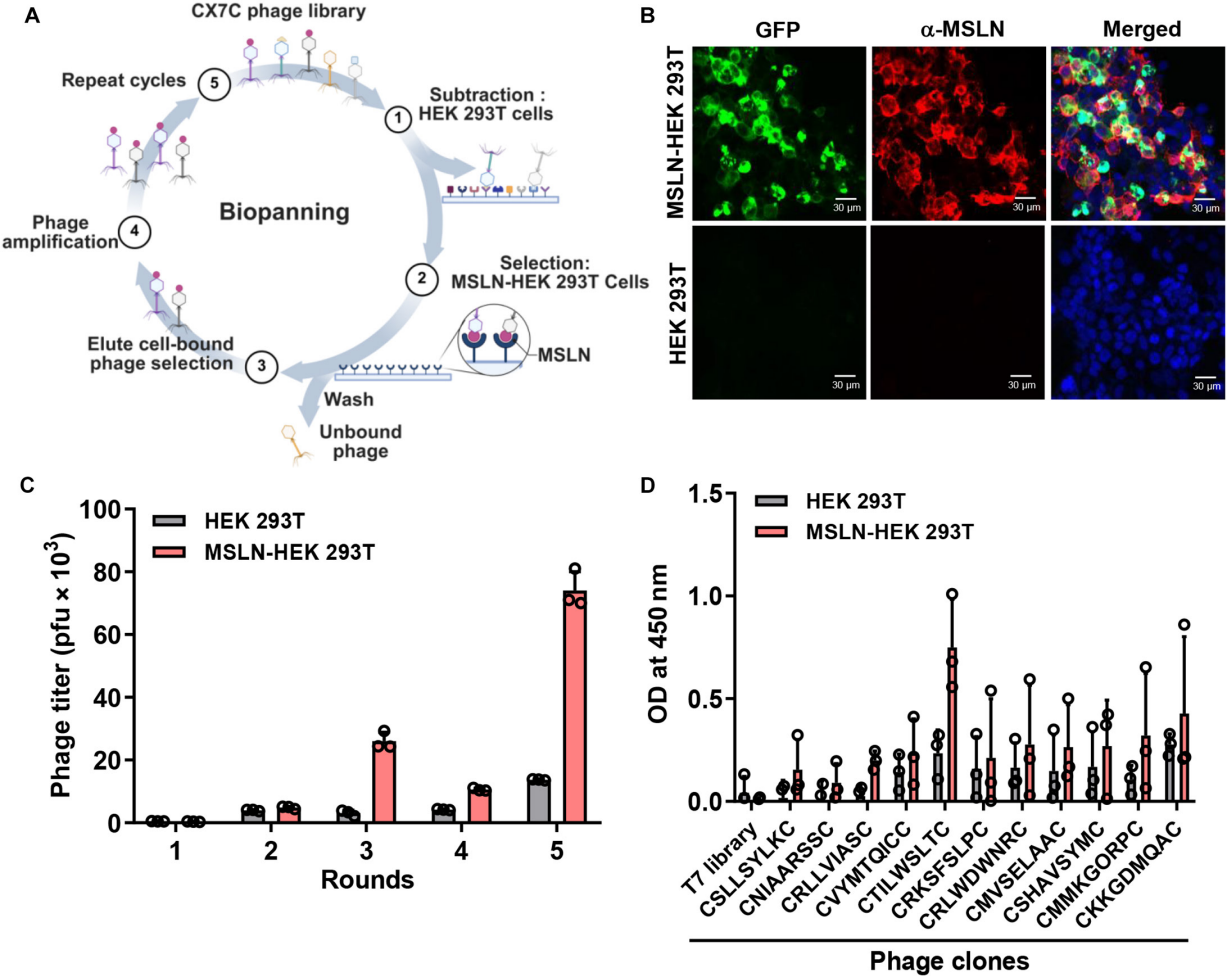
Biopanning of a phage library to identify mesothelin-binding peptides. (A) Schematic of biopanning. A phage library was incubated with parental HEK 293T cells; unbound phage was then incubated with HEK 293T cells transfected with a green fluorescent protein (GFP)-tagged mesothelin (MSLN) expression vector (MSLN-HEK 293T). Cell-bound phage was eluted and amplified for the next round. Created with BioRender.com. (B) MSLN-HEK 293T cells 48 h after transfection (green) and parental HEK 293T cells stained with an anti-MSLN antibody (red) and diamidino-2-phenylindole (DAPI) (blue). (C) Phage titer enrichment across screening rounds. pfu, plaque-forming units. (D) Phage-cell-binding ELISA showing binding of individual phage clones to parental versus MSLN-expressing HEK 293T cells. OD, optical density.

To identify an MSLN-binding peptide, we biopanned a CX7C phage library using HEK 293T cells transfected with a GFP-tagged human MSLN expression vector. Each screening round included negative selection on nontransfected HEK 293T cells followed by positive selection on transfected cells (Fig. 1A). MSLN expression in transfected cells was verified by GFP fluorescence microscopy and antibody staining (Fig. 1B). After 5 rounds, titers of phages binding transfected HEK 293T cells increased approximately 4-fold relative to round 1 (Fig. 1C). One hundred phage clones from rounds 3 to 5 were randomly selected and sequenced to determine the displayed peptide sequences. Eleven CX7C-pattern clones were tested by cell-binding ELISA on MSLN-expressing HEK 293T cells. Among them, a clone displaying the CTILWSLTC sequence bound transfected cells preferentially over nontransfected cells and exceeded the binding of other clones (Fig. 1D).

### Specificity and affinity of MSLNpep binding to MSLN

**Fig. 2.**
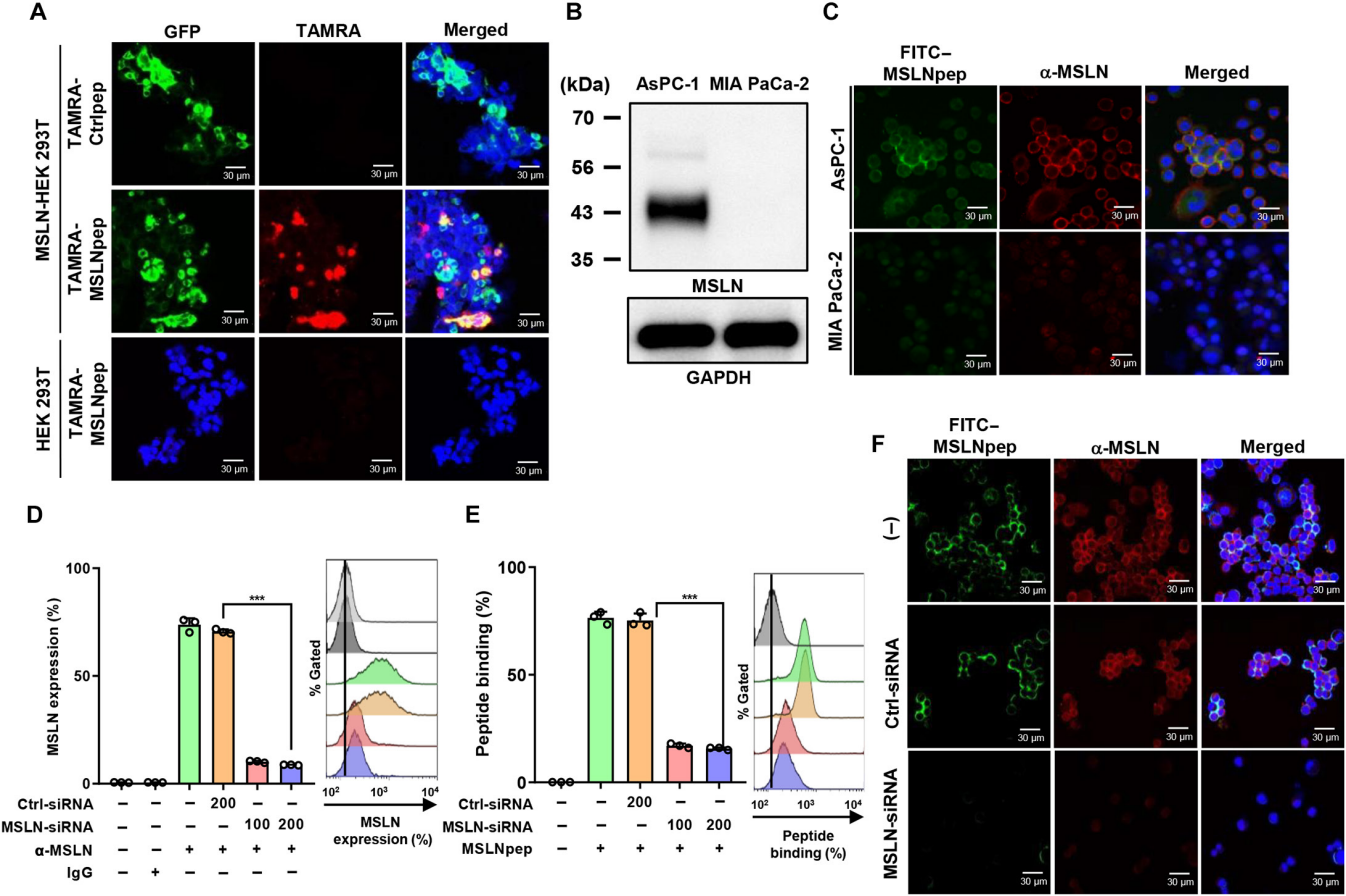
Binding of MSLNpep to MSLN-expressing cells. (A) Parental and GFP-tagged (green) MSLN-expressing HEK 293T cells were incubated with tetramethylrhodamine-5-maleimide (TAMRA)-labeled MSLNpep or a control peptide (red) at 4 °C for 1 h and DAPI (blue). (B) Immunoblotting of MSLN expression in AsPC-1 and MIA PaCa-2 pancreatic tumor cells. GAPDH, glyceraldehyde-3-phosphate dehydrogenase. (C) AsPC-1 and MIA PaCa-2 cells were incubated with fluorescein isothiocyanate (FITC)–MSLNpep (green) at 4 °C for 1 h and an anti-MSLN antibody (red). Nuclei were stained with DAPI (blue), and images were merged. (D and E) AsPC-1 cells were treated with small interfering RNA (siRNA) against MSLN (100 and 200 nM) to knock down MSLN expression or with control (Ctrl) siRNA (200 nM). Cells were incubated with an anti-MSLN antibody (D) or 25 μM FITC–MSLNpep (E) and analyzed by flow cytometry. ***P < 0.001 by one-way ANOVA. (F) AsPC-1 cells were treated with MSLN-siRNA or Ctrl-siRNA (200 nM). After silencing, cells were incubated with 25 μM FITC–MSLNpep (green) at 4 °C for 1 h and an anti-MSLN antibody (red) (1:1,000). Nuclei were stained with DAPI (blue), and images were merged. Scale bars, 30 μm.

The CTILWSLTC peptide was synthesized in cyclic form via a disulfide bond between the N- and C-terminal cysteines and designated MSLNpep. TAMRA-labeled MSLNpep bound to GFP-tagged MSLN-expressing HEK 293T cells but not to nontransfected cells, whereas a control peptide showed no binding (Fig. 2A). To examine binding to MSLN-high tumor cells, we assessed MSLN expression in human pancreatic tumor cell lines. AsPC-1, PANC-1, and BxPC-3 cells expressed higher MSLN levels than MIA PaCa-2 cells (Fig. S1 and Fig. 2B). Consistently, FITC-labeled MSLNpep bound AsPC-1 cells more strongly than MIA PaCa-2 cells and colocalized with MSLN in AsPC-1 cells (Fig. 2C). Knockdown of *MSLN* with small interfering RNA reduced MSLN expression and MSLNpep binding in AsPC-1 cells, as determined by flow cytometry (Fig. 2D and E) and immunofluorescence microscopy (Fig. 2F).

**Fig. 3.**
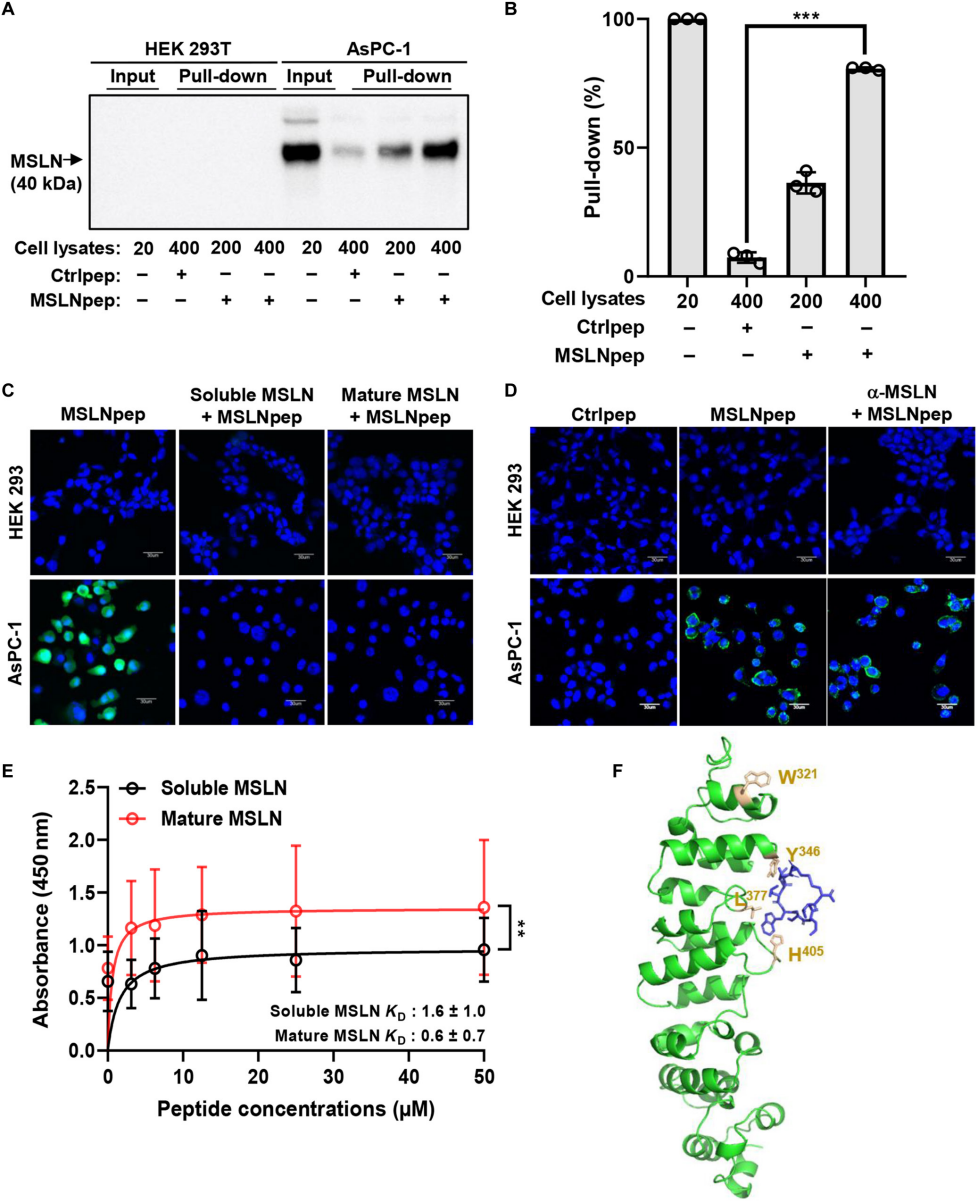
Binding of MSLNpep to MSLN protein. (A) HEK 293T and AsPC-1 cell lysates (20, 200, and 400 μg) were incubated with biotin-labeled MSLNpep or control peptide (Ctrlpep) and pulled down with streptavidin-coated magnetic beads. Immunoblotting of the precipitates was performed using an anti-MSLN antibody. (B) Quantification of band intensity (%) for each pull-down relative to input in (A) using ImageJ software. Data are mean ± standard error from 3 independent experiments. ***P < 0.001 by one-way analysis of variance (ANOVA). (C) HEK 293 and AsPC-1 cells were incubated at 4 °C for 1 h with FITC-labeled MSLNpep together with either soluble or mature MSLN. Nuclei were stained with DAPI (blue), and images were merged. Scale bars, 30 μm. (D) HEK 293 and AsPC-1 cells were pretreated with an anti-MSLN antibody at room temperature for 1 h and incubated with FITC–MSLNpep (green) at 4 °C for 1 h. Nuclei were stained with DAPI (blue), and images were merged. Scale bars, 30 μm. (E) ELISA quantifying MSLNpep binding to soluble and mature MSLN. Data are mean ± standard error from 3 independent experiments. **P < 0.01 by unpaired *t* test. (F) In silico modeling of MSLNpep binding to MSLN using the PEPFOLD program. MSLN, green; MSLNpep, blue.

To test whether MSLN mediates MSLNpep binding to AsPC-1 cells, cell lysates were incubated with biotin-labeled peptides and subjected to streptavidin pull-down and immunoblotting with an anti-MSLN antibody. Biotin-labeled MSLNpep, but not a control peptide, pulled down MSLN from AsPC-1 cells in a dose-dependent manner, whereas little pull-down was observed from nontransfected HEK 293T cells (Fig. 3A and B). Both mature (residues 296 to 606) and soluble (residues 296 to 592) forms of MSLN inhibited MSLNpep binding to AsPC-1 cells (Fig. 3C). In contrast, an anti-MSLN antibody did not inhibit MSLNpep binding (Fig. 3D). KPC mouse pancreatic tumor cells also expressed MSLN (Fig. S2A); however, MSLNpep did not bind these cells (Fig. S2B). These findings suggest that MSLNpep recognizes a shared epitope on the mature and soluble forms of human MSLN, but not a conserved epitope between human and mouse MSLN.

Next, we measured the binding affinity of MSLNpep for MSLN by ELISA. MSLNpep bound the mature form at higher levels than the soluble form (*K*_D_ = 0.6 μM vs 1.6 μM; Fig. 3E). For structure-based analysis, we performed docking of MSLNpep to MSLN (residues 207 to 600) using DiffDock [27]. Among the 10 top-ranked poses, 8 positioned MSLNpep within the hydrophobic groove spanning domains A and B and involving Tyr^346^, Leu^377^, and His^405^ of MSLN; this interaction was mediated by hydrophobic residues of MSLNpep (Ile^3^, Leu^4^, Trp^5^, and Leu^7^) (Fig. 3F). In addition, we performed a point-mutation analysis of MSLNpep by substituting each amino acid residue with alanine (alanine scan). Internalization assays using each mutant peptide labeled with a pH-sensitive dye that emits fluorescence at acidic pH showed that Ser^6^ and Thr^8^ as well as the hydrophobic residues (Trp^5^ and Lue^7^) of MSLNpep were key determinants for MSLNpep internalization into AsPC-1 cells (Fig. S3).

### MSLNpep inhibits the migration and invasion of MSLN-high tumor cells

**Fig. 4.**
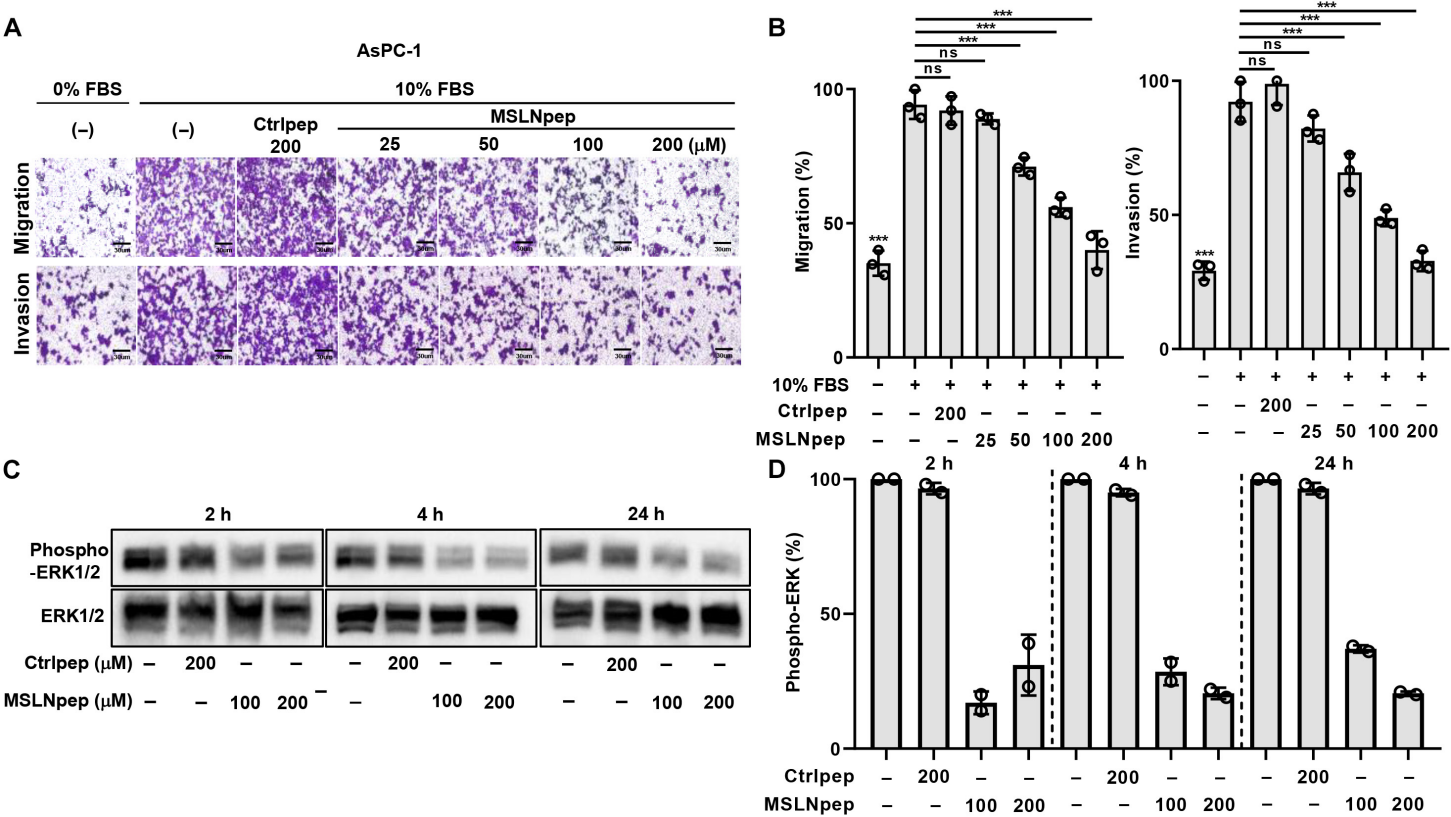
Inhibition of tumor cell migration and invasion by MSLNpep. (A) Upper chambers with uncoated (migration) or Matrigel-coated (invasion) membranes were seeded with AsPC-1 cells and incubated in medium containing either Ctrlpep or MSLNpep at the indicated concentrations for 16 to 18 h; lower chambers contained medium with 0% or 10% fetal bovine serum (FBS). Scale bars, 30 μm. (B) Cells that migrated through the membrane or invaded the Matrigel in (A) were counted in ImageJ. Migration and invasion are expressed as a percentage of the peptide-untreated 10% FBS control. Data are mean ± standard error from 3 independent experiments. ***P < 0.001; ns, not significant by one-way ANOVA. (C) AsPC-1 cells were incubated with 10% FBS and Ctrlpep (200 μM) or MSLNpep (100 or 200 μM) for 2, 4, or 24 h. Cell lysates were immunoblotted with anti-phospho-ERK1/2 and anti-ERK1/2 antibodies. (D) Band intensities for phospho-ERK1/2, normalized to total ERK1/2, were quantified in ImageJ. Values are expressed as a percentage of the peptide-untreated group. Data are mean ± standard error from 3 independent experiments.

MSLN regulates cell migration and invasion, with ERK phosphorylation as a key downstream signal [32]. MSLNpep treatment inhibited AsPC-1 cell migration (wound closure) and invasion in a dose-dependent manner (Fig. 4A and B; Fig. S4A and B) and similarly inhibited PANC-1 cells (Fig. S5A and B). The ratio of phosphorylated ERK to total ERK decreased as early as 2 h and remained reduced up to 24 h after MSLNpep treatment (Fig. 4C and D). These data indicate that MSLNpep binds MSLN on tumor cells and suppresses migration and invasion by reducing ERK phosphorylation.

### MSLN-targeted mitochondrial-membrane-damaging peptides induce selective cytotoxicity in MSLN-high tumor cells

**Fig. 5.**
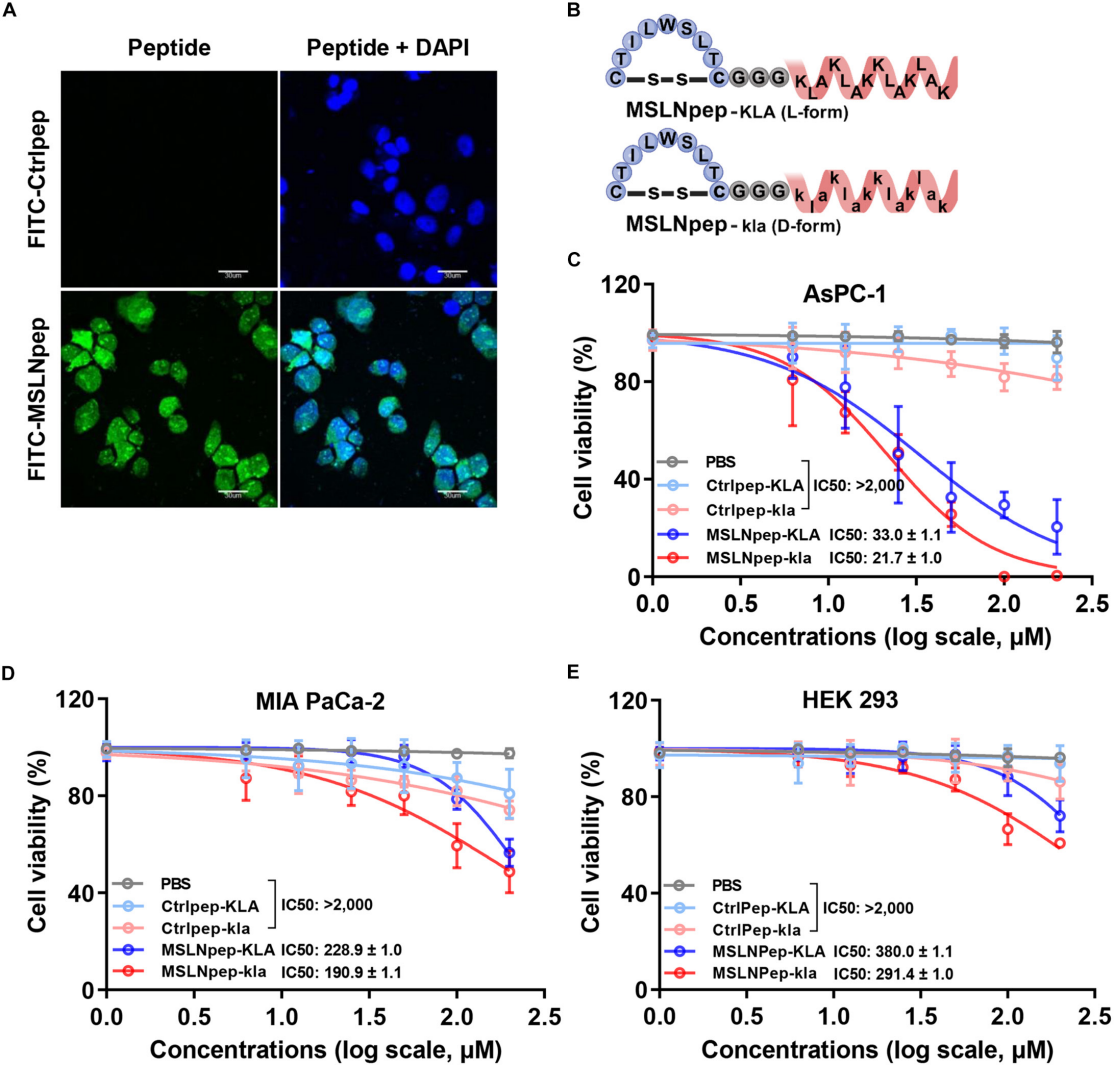
Cytotoxicity of an MSLN-targeted mitochondrial-membrane-damaging peptide. (A) AsPC-1 cells were incubated at 37 °C for 2 h for 1 h with 25 μM FITC-labeled control peptide (Ctrlpep) or MSLNpep (green). Nuclei were stained with DAPI (blue), and images were merged. Scale bars, 30 μm. (B) Diagram of MSLNpep-KLA (L-form) and MSLNpep-kla (D-form). (C to E) AsPC-1 (C), MIA PaCa-2 (D), and HEK 293T (E) cells were incubated with phosphate-buffered saline (PBS), Ctrlpep-KLA, Ctrlpep-kla, MSLNpep-KLA, or MSLNpep-kla for 24 h. After incubation, cell viability was measured using Cell Counting Kit reagents. The half-maximal inhibitory concentration (IC50) values are presented as mean ± standard error from 3 independent experiments. Created with BioRender.com.

We tested whether MSLNpep delivers therapeutic agents into MSLN-high tumor cells. FITC-labeled MSLNpep efficiently internalized into MSLN-high AsPC-1 (Fig. 5A) and PANC-1 cells (Fig. S6) after 2 h of incubation, indicating that MSLNpep is a useful tool for cytoplasmic drug delivery in MSLN-high tumors. As MSLN-targeted therapeutics, we synthesized chimeric peptides linking MSLNpep to mitochondrial-membrane-damaging peptides: the L-form (KLAKLAK)2 and the D-form (klaklak)2 (MSLNpep-KLA and MSLNpep-kla, respectively; Fig. 5B). Both MSLNpep-kla and MSLNpep-KLA induced greater cell death in MSLN-high AsPC-1 cells than control peptides (Fig. 5C). MSLNpep-kla was more cytotoxic than MSLNpep-KLA (half-maximal inhibitory concentration = 21.7 μM vs 33 μM; Fig. 5C). Relative to AsPC-1 cells, both conjugates showed lower cytotoxicity in MSLN-low MIA PaCa-2 (Fig. 5D) and HEK 293T cells (Fig. 5E). Thus, MSLNpep mediates selective delivery of mitochondrial-membrane-damaging peptides to MSLN-high tumor cells, with greater sensitivity to MSLNpep-kla than to MSLNpep-KLA.

### MSLN-targeted mitochondrial-membrane-damaging peptides inhibit pancreatic tumor growth in mice

To assess in vivo tumor homing, we intravenously injected TAMRA-labeled MSLNpep into mice bearing subcutaneous AsPC-1 tumors and collected organs 6 h later. MSLNpep accumulated in AsPC-1 tumors at higher levels than in off-target organs (liver, lung, and kidney; Fig. S7A). In addition, when peptide localization relative to blood vessels was examined, MSLNpep was found to localize outside blood vessels within tumor tissues (Fig. S7B). These results suggest that MSLNpep can serve as a ligand to selectively deliver therapeutic agents to tumor tissues while sparing the liver and other organs.

**Fig. 6.**
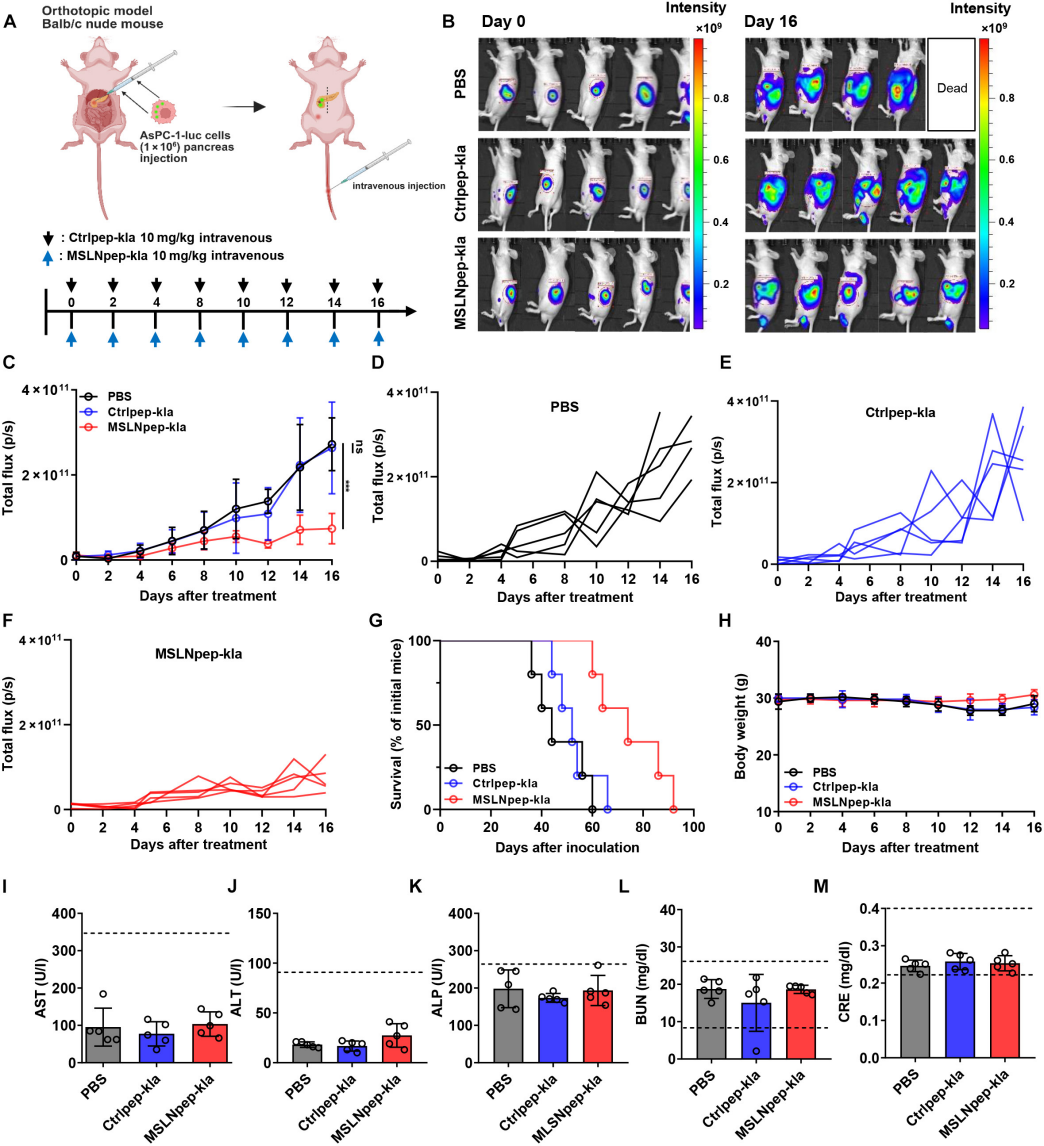
Inhibition of orthotopic pancreatic tumor growth by an MSLN-targeted mitochondrial-membrane-damaging peptide. (A) Treatment schema for mice bearing orthotopic AsPC-1-luc pancreatic tumors. Mice received PBS, Ctrlpep-kla, or MSLNpep-kla at 10 mg/kg body weight. (B) Whole-body bioluminescence imaging on days 0 and 16 after treatment. (C) Total bioluminescent flux after treatment. Data are mean ± standard error (*n* = 5). ***P < 0.001; ns, not significant by 2-way ANOVA. (D to F) Total bioluminescent flux for each mouse after treatment with PBS (D), Ctrlpep-kla (E), or MSLNpep-kla (F). (G) Survival rates. (H) Body weights. (I) Serum aspartate aminotransferase (AST) levels. (J) Serum alanine aminotransferase (ALT) levels. (K) Serum alkaline phosphatase (ALP) levels. (L) Serum blood urea nitrogen (BUN) levels. (M) Serum creatinine (CRE) levels. Created with BioRender.com.

To evaluate the antitumor activity of MSLN-targeted mitochondrial-membrane-damaging peptides, we established an orthotopic pancreatic tumor model by surgically injecting luciferase-expressing AsPC-1 cells into the pancreas of nude mice, then administered MSLNpep-kla systemically via the tail vein (Fig. 6A). Tumor burden was monitored by intraperitoneal D-luciferin followed by whole-body bioluminescence imaging (Fig. 6B). Compared with saline and control-kla, MSLNpep-kla significantly suppressed bioluminescence-based tumor growth (Fig. 6B to F) and prolonged survival (Fig. 6G). Body weight remained stable during treatment (Fig. 6H). Serum liver enzymes (aspartate aminotransferase, alanine aminotransferase, and alkaline phosphatase) and kidney markers (blood urea nitrogen and creatinine) remained within reference ranges after treatment, indicating no apparent hepatotoxicity or nephrotoxicity (Fig. 6I to M).

We also tested MSLNpep-KLA. In serum-stability assays, MSLNpep-KLA showed minimal degradation for up to 12 h (Fig. S8). In mice bearing orthotopic AsPC-1-luc tumors, systemic administration of MSLNpep-KLA inhibited tumor growth compared with controls (Fig. S9A to F) and prolonged survival (Fig. S9G) without affecting body weight (Fig. S9H). Similarly, in a subcutaneous AsPC-1 model, MSLNpep-KLA inhibited tumor growth compared with controls (Fig. S10A). Body weight and serum liver and kidney markers remained unchanged during treatment with MSLNpep-KLA (Fig. S10B to G). Histology showed increased tumor cell death after MSLNpep-KLA treatment (Fig. S10H).

### MSLN-targeted mitochondrial-membrane-damaging peptide inhibits tumor growth in pancreatic cancer PDOs

**Fig. 7.**
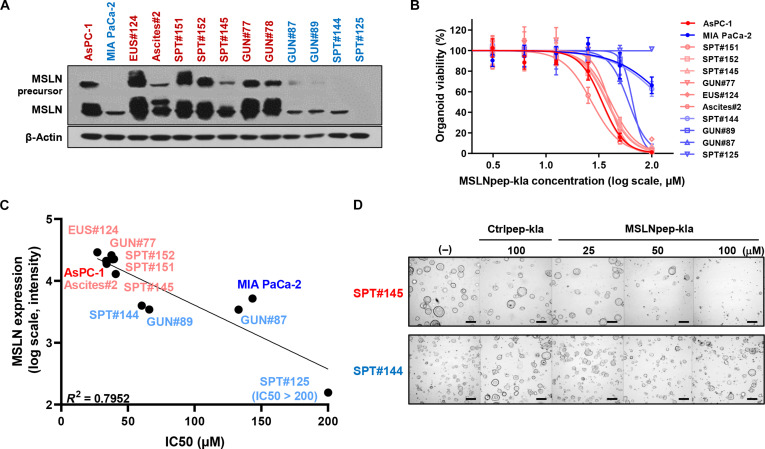
Cytotoxicity of an MSLN-targeted mitochondrial-membrane-damaging peptide in human pancreatic cancer patient-derived organoids (PDOs). (A) Immunoblot analysis of MSLN levels in human pancreatic cancer PDOs. Red, MSLN-high organoid; blue, MSLN-low organoid. AsPC-1 and MIA PaCa-2 cells served as controls. (B) Cytotoxicity of MSLNpep-kla in human pancreatic cancer PDOs. Red, MSLN-high organoid; blue, MSLN-low organoid. (C) Correlation between MSLN levels and IC50 values in PDOs (*R*^2^ = 0.7952). (D) Microscopic morphology of SPT#145 and SPT#144 organoids after treatment with Ctrlpep-kla (100 μM) or MSLNpep-kla (25, 50, and 100 μM). Scale bars, 200 μm.

To evaluate the potential of MSLN-targeted mitochondrial-membrane-damaging peptides for clinical translation, we assessed the cytotoxic efficacy of MSLNpep-kla in human pancreatic cancer PDOs. Western blotting showed heterogeneous MSLN expression across 11 PDO lines, regardless of TNM stage, enabling classification into MSLN-high and MSLN-low groups (Fig. 7A). Notably, MSLNpep-kla treatment induced strong cytotoxicity specifically in MSLN-high organoids while exhibiting minimal cytotoxicity in MSLN-low organoids (Fig. 7B). Cytotoxicity correlated with MSLN expression (*R*^2^ = 0.7952; Fig. 7C), supporting this selectivity. Microscopy further showed that MSLNpep-kla was more cytotoxic in MSLN-high organoids (e.g., SPT#145) than in MSLN-low organoids (e.g., SPT#144), whereas control peptide-kla produced negligible cytotoxicity irrespective of MSLN expression (Fig. 7D). These data indicate that MSLNpep-kla preferentially targets MSLN-high PDOs and efficiently delivers cytotoxic payloads. Selective cytotoxicity was independent of tumor stage, supporting applicability across clinical contexts. Overall, MSLNpep-kla is a potent, selective candidate therapy for MSLN-expressing pancreatic tumors with clear translational potential.

### Validation of MSLN as a specific therapeutic target in pancreatic cancer using scRNA-seq analysis

**Fig. 8.**
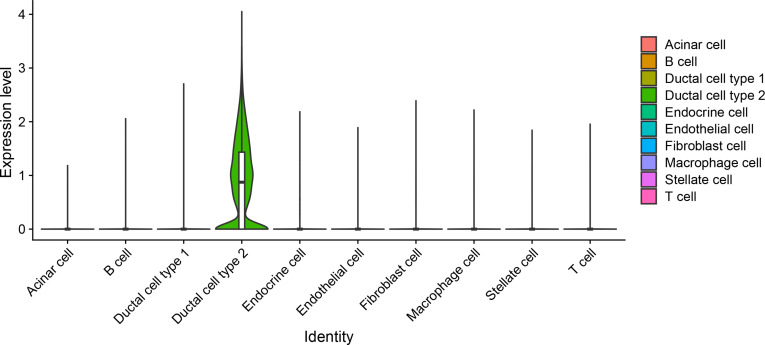
Single-cell expression analysis of *MSLN* in human pancreatic cancer tissues. Violin plots showing the expression distribution of *MSLN* across various cell types identified within the pancreatic cancer tumor microenvironment (dataset from Ellerby et al. [33]). The *y*-axis represents log-normalized expression levels.

To validate MSLN as a precise therapeutic target and assess potential off-tumor expression, we analyzed a large-scale scRNA-seq dataset of human pancreatic cancer tissues [31]. MSLN expression was predominantly restricted to the malignant ductal cell population (ductal cell type 2); in contrast, negligible expression was observed in normal ductal cells (ductal cell type 1) as well as in tumor microenvironment components, including immune cells (T cells, B cells, and macrophages), fibroblasts, and endothelial cells, indicating high tumor specificity (Fig. 8). Furthermore, quantitative analysis revealed a significant positive correlation between *MSLN* and the epithelial marker *KRT19* within the ductal cell clusters (Spearman’s *r* = 0.58, *P* < 2.2 × 10^−16^), proving that MSLN is specifically enriched in the malignant epithelial compartment of pancreatic cancer. These results support the rationale that MSLNpep can selectively target cancer cells while minimizing potential toxicity to normal tissues.

## Discussion

In this study, MSLNpep, an MSLN-binding peptide, was identified from a phage-displayed peptide library. A synthetic cyclic form of MSLNpep inhibited tumor-cell migration and invasion and selectively delivered mitochondrial-membrane-damaging peptides to pancreatic tumor cells by binding MSLN on the tumor-cell surface (Fig. S11A and B). When linked to mitochondrial-membrane-damaging peptides, (KLAKLAK)2 and (klaklak)2, MSLNpep mediated selective internalization of the chimeras into MSLN-high tumor cells, induced cell death, and, in mice, inhibited tumor growth without significant hepatotoxicity or nephrotoxicity. The D-form enantiomer (klaklak)2 is more resistant to peptidase degradation than the L-form (KLAKLAK)2 and disrupts mitochondrial membranes in a chirality-independent manner [34,35]. In support of this, (klaklak)2 conjugated to an integrin αvβ3-targeting RGD peptide inhibited tumor growth in mice [33]. Notably, MSLNpep-kla exerted higher cytotoxicity and more effectively inhibited tumor growth in mice than MSLNpep-KLA. MSLNpep-kla also induced tumor cell death in human pancreatic cancer PDOs, supporting its translational potential.

The amphiphilic (KLAKLAK)2 peptide, originally developed as an antibacterial peptide, damages mitochondrial membranes, releases cytochrome *c*, and induces cell death [36,37]. Because (KLAKLAK)2 causes minimal damages to the plasma membrane of mammalian cells, the mechanism by which it escapes from the endosomes and ultimately targets mitochondria remains unclear. However, when locally concentrated within endosomes via ligand (e.g., MSLNpep)-mediated endocytosis, (KLAKLAK)2 may damage the endosomal membrane, allowing a fraction of the peptide to escape from endosomes [38,39].

MUC16, a membrane protein and MSLN-binding ligand, contributes to mucus composition, barrier protection, and glycosylation. Its extracellular domain is enriched in repeats such as Gly-Ser-Thr. Sequence analysis revealed no homology between MUC16 and MSLNpep, indicating that MSLNpep engages an MSLN epitope distinct from that recognized by MUC16. Peptides selected against human proteins can cross-react with the murine ortholog; however, MSLNpep did not bind KPC mouse pancreatic tumor cells that highly express mouse MSLN. This finding implies interspecies differences in the MSLN epitope recognized by MSLNpep. Binding of MSLNpep to MSLN-positive tumor cells was impeded by both soluble (shed) and mature MSLN, yet MSLNpep bound mature MSLN with higher affinity. Collectively, MSLNpep is expected to preferentially engage mature MSLN on tumor-cell surfaces within the tumor microenvironment, where soluble MSLN acts as a competitive inhibitor.

Among antibody-based MSLN therapies, the immunotoxin SS1P comprises an anti-MSLN single-chain Fv fused to *Pseudomonas* exotoxin A [8]. After binding MSLN, SS1P is internalized and inhibits elongation factor-2, thereby blocking protein synthesis and inducing cell death [40]. However, SS1P can cause capillary leak syndrome and pleuritis via inflammatory injury to vascular endothelium and normal pleural mesothelium, respectively [41]. It is also taken up by proximal tubular cells, causing renal injury and albuminuria [42]. Another MSLN-targeted antibody approach is the antibody–drug conjugate anetumab ravtansine, which couples an anti-MSLN antibody to the microtubule inhibitor DM4; upon internalization into MSLN-expressing tumor cells, DM4 is released and kills the cells effectively [16]. Moreover, MSLN-targeted CAR-T cells have been evaluated in pancreatic, ovarian, and triple-negative breast cancers [18,19]. In a phase I clinical trial in pancreatic cancer, an MSLN-targeted CAR-T therapy improved hepatic lesions but had no effect on the primary tumor [43]. In another phase I clinical trial in malignant pleural disease, combining CAR-T cells with the anti-PD-1 antibody pembrolizumab increased median overall survival [44]. CAR-T cells expressing monoclonal antibody 15b, which targets an epitope proximal to the membrane cleavage site, inhibited tumor growth in mice more efficiently than CAR-T cells recognizing an epitope within shed or soluble MSLN [45].

To date, there are no Food and Drug Administration-approved therapeutic agents that specifically target MSLN, highlighting the need for novel therapeutic tools [46,47]. Compared to normal tissue, the extracellular matrix of tumor, termed oncomatrix, exhibits the increased accumulation of matrix proteins including collagen, leading to fibrotic stiffness and impaired oxygen diffusion (hypoxia) within the tumor microenvironment [48]. In addition, the pH of the tumor microenvironment is lower than that of normal tissues (pH 6.8 to 7.0 vs 7.4), which enhances tumor-cell invasiveness, reduces the activity of chemotherapeutic agents, and promotes chemoresistance [49,50]. The 9-mer MSLNpep is expected to penetrate fibrotic stromal tissue in pancreatic cancer more efficiently than antibodies, indicating its promise as a ligand for MSLN-targeted drug delivery. Additionally, mitochondria generate the energy required for cell survival; accordingly, tumor cells may be less likely to develop resistance to mitochondrial-membrane-disrupting agents such as MSLNpep-kla. A mesothelin-deficient mouse exhibited no significant phenotypic change, suggesting that modulating MSLN is unlikely to produce major adverse physiological effects in patients. MSLNpep-kla caused no detectable hepatic or renal toxicity in mice, supporting its potential clinical safety. Future work should conjugate MSLNpep to radioisotopes for peptide receptor radionuclide therapy or to chemotherapeutic agents to generate peptide–drug conjugates for MSLN-targeted cancer therapy.

## Data Availability

The data are freely available upon request.
