# Supplementary material for: Mesothelin-Binding Peptide Inhibits Cell Migration and Enables Targeted Delivery of a Mitochondrial-Membrane-Damaging Peptide to Pancreatic Tumors
Source: Biomater Res. 2026 May 11;30:0361. doi: 10.34133/bmr.0361 (PMC13158456; doi:10.34133/bmr.0361)
Supplement: Supplementary 1 — Supplementary Methods Figs. S1 to S11 [file bmr.0361.f1.doc]

**SUPPLEMENTARY MATERIALS**

**Supplementary Methods**

**Point mutational analysis of peptide internalization**

To examine the internalization of point-mutated MSLNpep, each amino acid residue at positions 2–8 of MSLNpep was mutated to alanine (alanine scan) and labeled with a pH-sensitive dye, which emits red fluorescence at acidic pH. Cells were incubated at 37 ℃ for 2 h with 25 µM wild-type and mutant peptides, including Thr^2^-to-Ala (T2A), Ile^3^-to-Ala (I3A), Leu^4^-to-Ala (L4A), Trp^5^-to-Ala (W5A), Ser^6^-to-Ala (S6A), Leu^7^-to-Ala (L7A), and Thr^8^-to-Ala (T8A). Nuclei were stained with DAPI, and images were merged.

**Scratch (wound closure) assays**

AsPC-1 cells were seeded in culture plates and grown to near confluence in RPMI-1640 supplemented with 10% FBS, 100 U/mL penicillin, and 100 μg/mL streptomycin. Cells were maintained at 37 °C in a humidified incubator with 5% CO_2_. Cells were serum-starved in medium containing 1% FBS for 6 h, and a linear scratch was generated using a sterile 200 µL pipette tip. After washing with PBS to remove debris, cells were incubated in medium containing the indicated concentrations of peptides in the presence of 10% FBS. Images of the wound area were captured every 4 h for up to 24 h, and wound closure was quantified using the ImageJ program.

**Tumor homing assays and liver and kidney function tests in mice bearing tumor**

When subcutaneous AsPC-1 tumors reached approximately 150 mm³, mice received an intravenous injection of a TAMRA-labeled peptide (10 mg/kg body weight). TAMRA was chosen because it exhibits lower background fluorescence in tissues than FITC. After 6 h of circulation, mice were euthanized, and the tumor and control organs (kidney, liver, and lung) were harvested. Frozen tissue sections were incubated with anti-MSLN and anti-CD31 antibodies (Abcam, Cambridge, UK) and imaged using a confocal microscope (Nanoscope Systems, Daejeon, Korea).

When tumors reached approximately 100 mm^3^ in size, tumor-bearing mice were injected via the tail vein with peptides (10 mg/kg body weight, three times per week for 3 weeks). At the end of treatment, blood was collected for analysis of serum liver enzymes (aspartate transferase [AST], alanine transferase [ALT], and alkaline phosphatase [ALP]) and kidney function markers (blood urea nitrogen [BUN] and creatinine [CRE]) by DGMIF (Daegu, Korea).

**Serum stability assays**

Mouse blood was collected and allowed to clot in a tube for 15–20 min at room temperature. Samples were centrifuged at 12,000 rpm at 4 °C, and the supernatant was collected. Peptides were incubated with the supernatant (serum) at a 50:50 (v/v) ratio for the indicated times. Samples were diluted 100-fold and fractionated by C18 reversed-phase high-performance liquid chromatography (HPLC) using a linear acetonitrile gradient (0.1% trifluoroacetate in water for equilibration; 0.1% trifluoroacetate in acetonitrile for elution) for peptide peak analysis.

**Supplementary Figures**

**
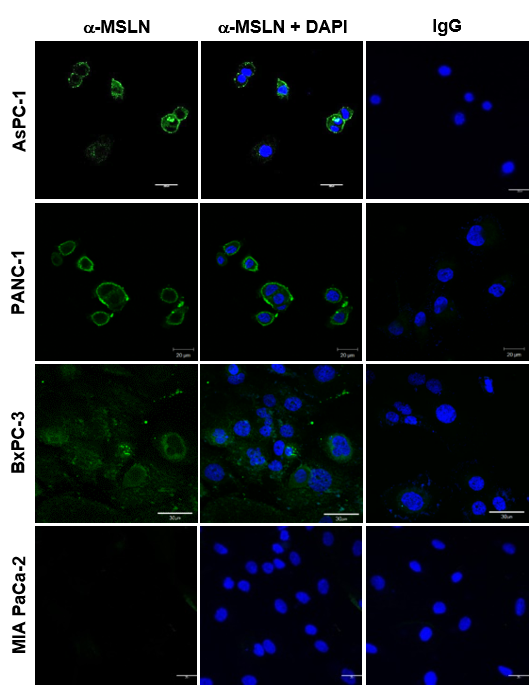
**

**Figure S1. MSLN expression in human pancreatic tumor cells.** Pancreatic tumor cell lines (AsPC-1, PANC-1, BxPC-3, and MIA PaCa-2) were incubated with an IgG control and anti-MSLN antibody (green). Nuclei were stained with DAPI (blue), and images were merged. Scale bars: 30 μm.

**
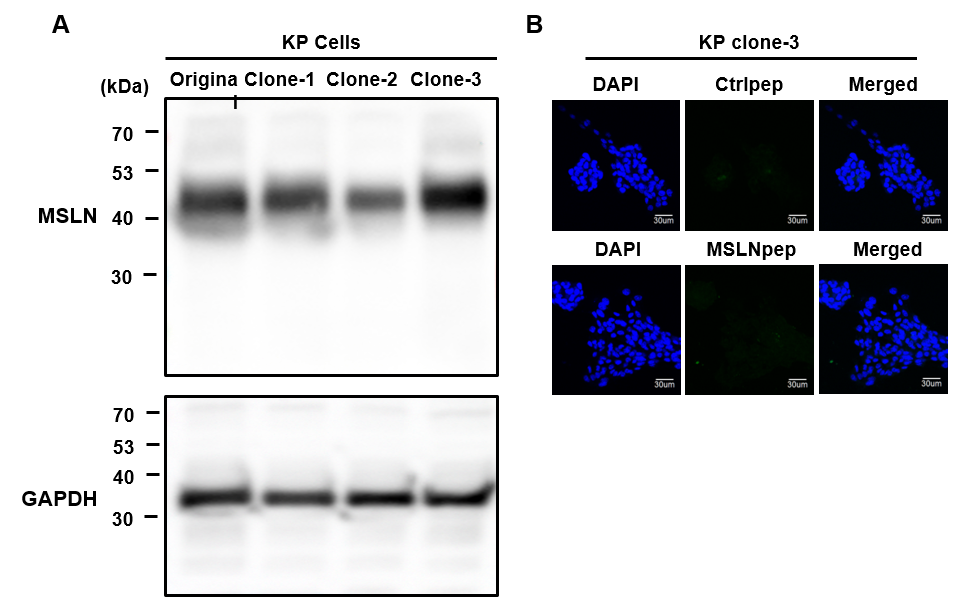
**

**Figure S2. Mouse MSLN expression in KP cells. (A)** Lysates of mouse KP cells (original, clone-1, clone-2, and clone-3) were immunoblotted with an anti–mouse MSLN antibody. GAPDH was used as a loading control. **(B)** Mouse KP clone-3 cells were incubated with FITC–MSLNpep (green). Nuclei were stained with DAPI (blue), and images were merged. Scale bars: 30 μm.


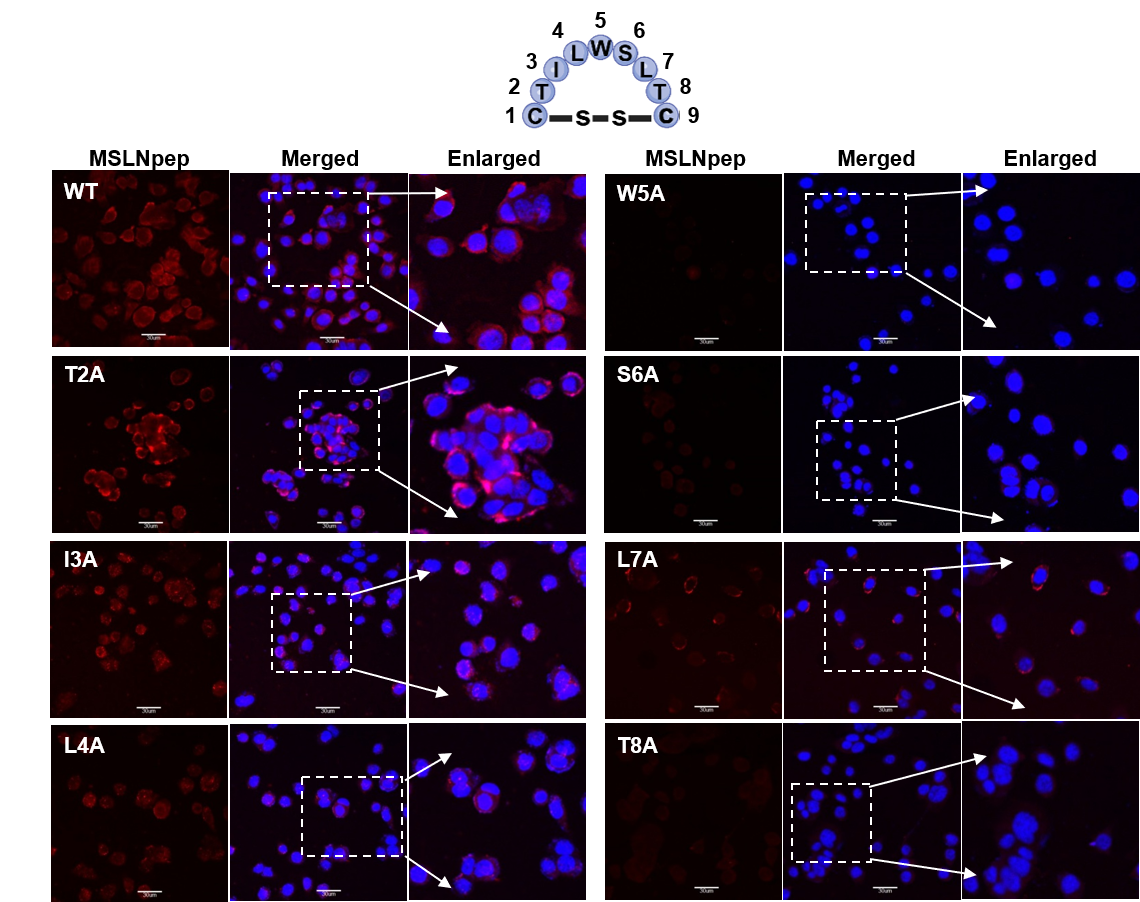


**Figure S3. Point mutational analysis of MSLNpep internalization into AsPC-1 cells.** Each amino acid at residue at positions 2–8 of MSLNpep was mutated to alanine (alanine scan) and labeled with a pH-sensitive dye that emits fluorescence at acidic pH to examine the internalization. Cells were incubated with 25 µM wild-type (WT) and mutant peptides (T2A, I3A, L4A, W5A, S6A, L7A, and T8A) at 37 ℃ for 2 h. Nuclei were stained with DAPI, and images were merged. Boxes indicate the enlarged areas. Scale bars: 30 μm.

**
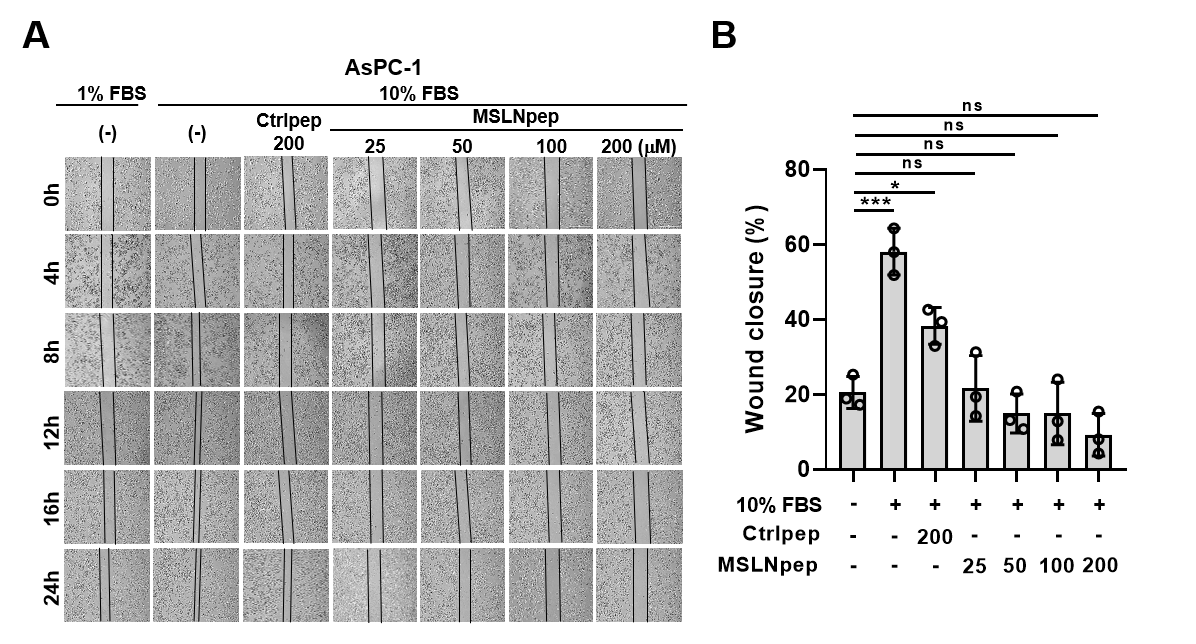
**

**Figure S4.** **Inhibition of cell migration and wound closure by MSLNpep.** (A) Representative images of cell migration and wound closure. AsPC-1 cells were incubated with MSLNpep (25, 50, 100, or 200 µM) and Ctrlpep (200 µM) in the presence of 10% FBS for the indicated time points after scratching. The 1% FBS group was used as a control. (B) Quantification of the percent wound closure after 24 h incubation. Data are presented as mean ± standard error from three independent experiments. *, *P* < 0.05, ***, *P* < 0.001; ns, significant as determined by one-way ANOVA.


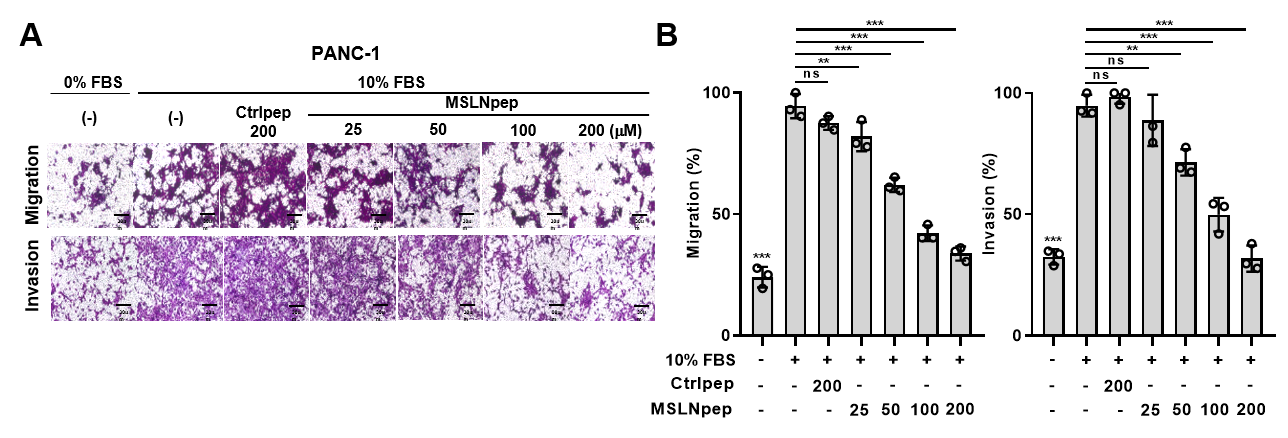


**Figure S5. Inhibition of PANC-1 cell migration and invasion by MSLNpep. (A)** Upper chambers with uncoated membranes (migration) or Matrigel-coated membranes (invasion) containing PANC-1 cells were incubated in medium with control peptide (Ctrlpep) or MSLNpep at the indicated concentrations for 16–18 h; lower chambers contained medium with 0% or 10% FBS. Scale bars: 30 μm. **(B)** Cells that migrated to the membrane or invaded Matrigel in (A) were counted in ImageJ. Percent migration and invasion relative to the peptide-untreated group with 10% FBS were calculated. Data are mean ± standard error from three independent experiments. **, *P* < 0.01; ***, *P* < 0.001; ns, not significant by one-way ANOVA.

**
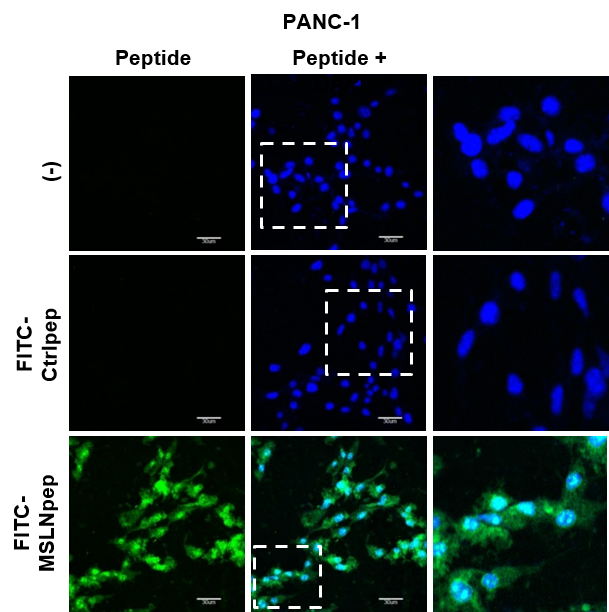
**

**Figure S6.** **Internalization of MSLNpep into PANC-1 cells**. PANC-1 cells were incubated with 25 µM FITC-labeled control peptide (Ctrlpep) and MSLNpep (green) at 37 °C for 2 h. Nuclei were stained with DAPI (blue), and images were merged. Right panels show enlarged views of boxed regions. Scale bars: 30 μm.

**
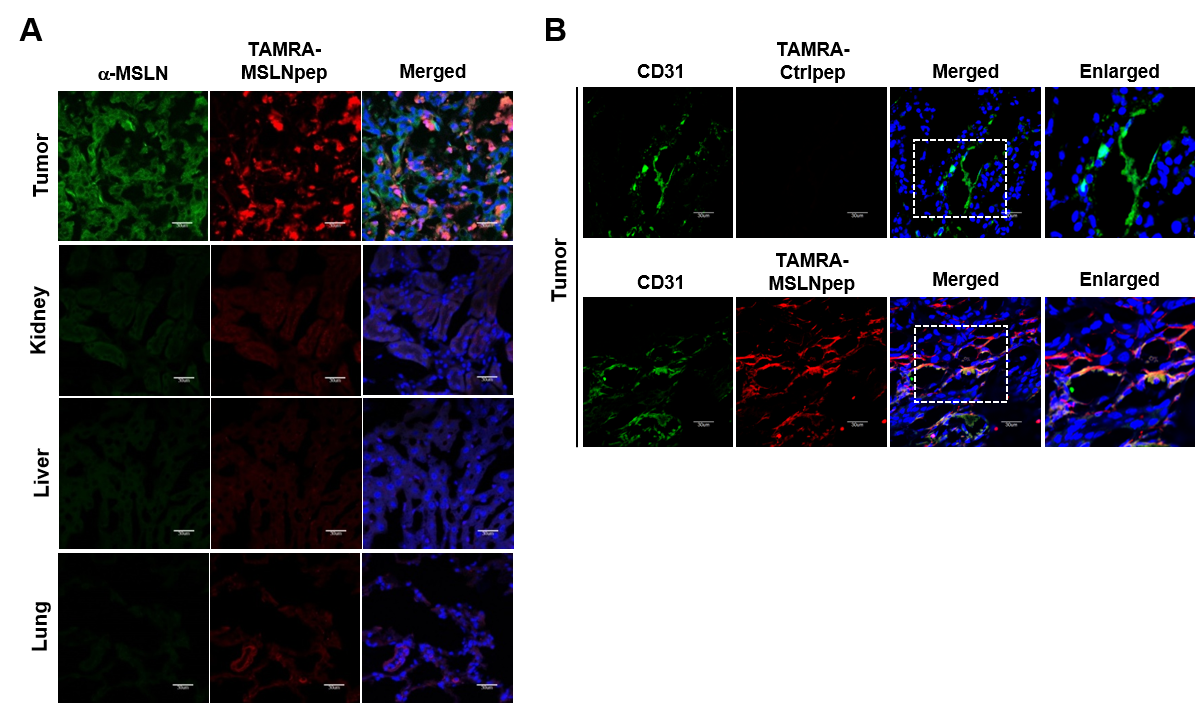
**

**Fig. S7. Tumor homing of MSLNpep.** Mice bearing subcutaneous AsPC-1 tumors received TAMRA-labeled MSLNpep or Ctrlpep (red) via the tail vein and were allowed to circulate for 6 h. (A) Frozen tumor and control organ sections were incubated with an anti-MSLN antibody (green). (B) Tumor sections were incubated with an anti-CD31 antibody (green) to visualize the vasculature. Nuclei were stained with DAPI (blue), and the images were merged. Boxes indicate the enlarged area. Scale bars: 30 μm.

**
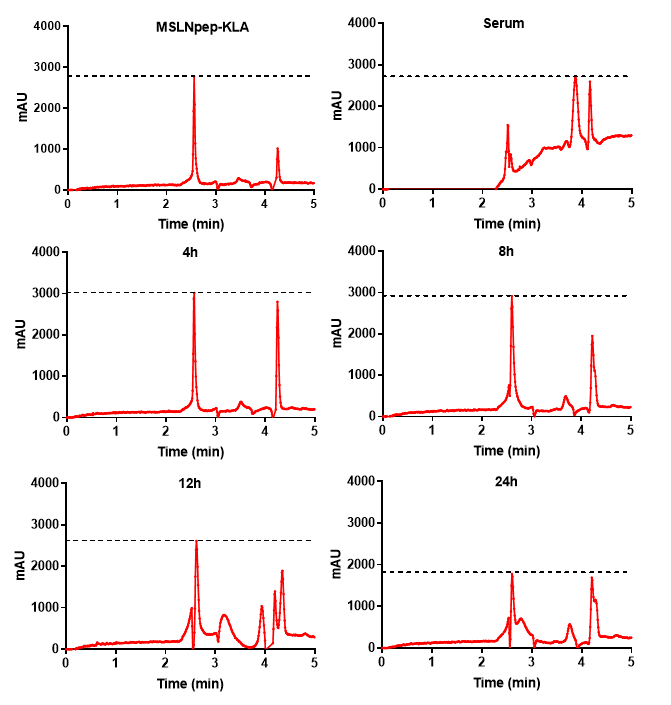
**

**Figure S8. Serum stability of MSLNpep–KLA peptide.** MSLNpep–KLA was incubated with serum for the indicated times, and peptide peaks were analyzed by LC–MS.

**
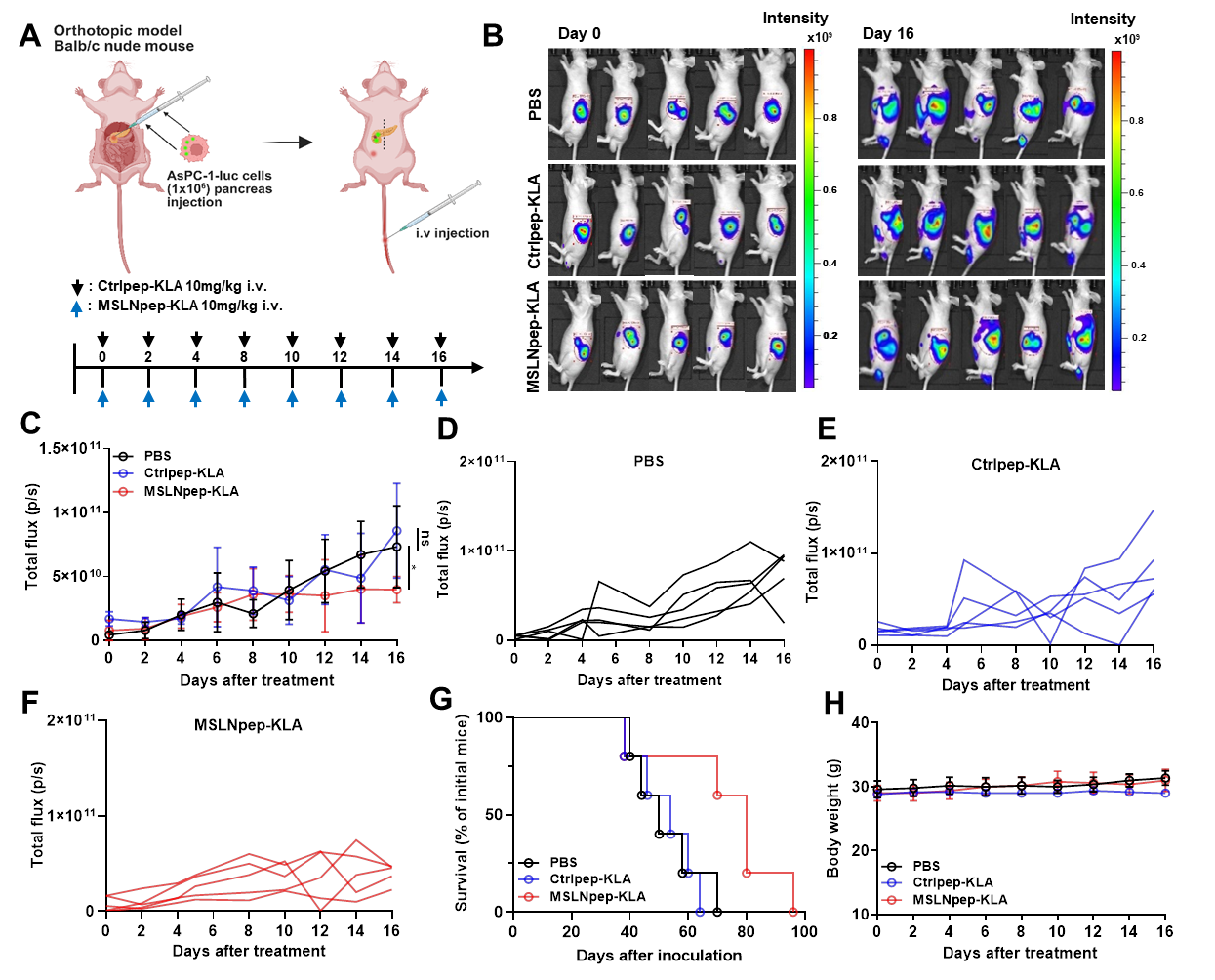
**

**Figure S9. Inhibition of orthotopic pancreatic tumor growth in mice by MSLN–KLA.** **(A)** Treatment schema for mice bearing orthotopic AsPC-1–luc pancreatic tumors. Mice received phosphate-buffered saline (PBS), control peptide (Ctrlpep)–KLA, or MSLNpep–KLA. **(B)** Whole-body bioluminescence imaging at days 0 and 16 after treatment. **(C)** Total bioluminescence flux after treatment. Data are mean ± standard error (n = 5). *, *P* < 0.05; ns, significant by two-way ANOVA. **(D–F)** Total bioluminescence flux for each mouse after treatment with PBS (D), Ctrlpep–KLA (E), or MSLNpep–KLA (F). **(G)** Survival. **(H)** Body weights. Created with BioRender.com.


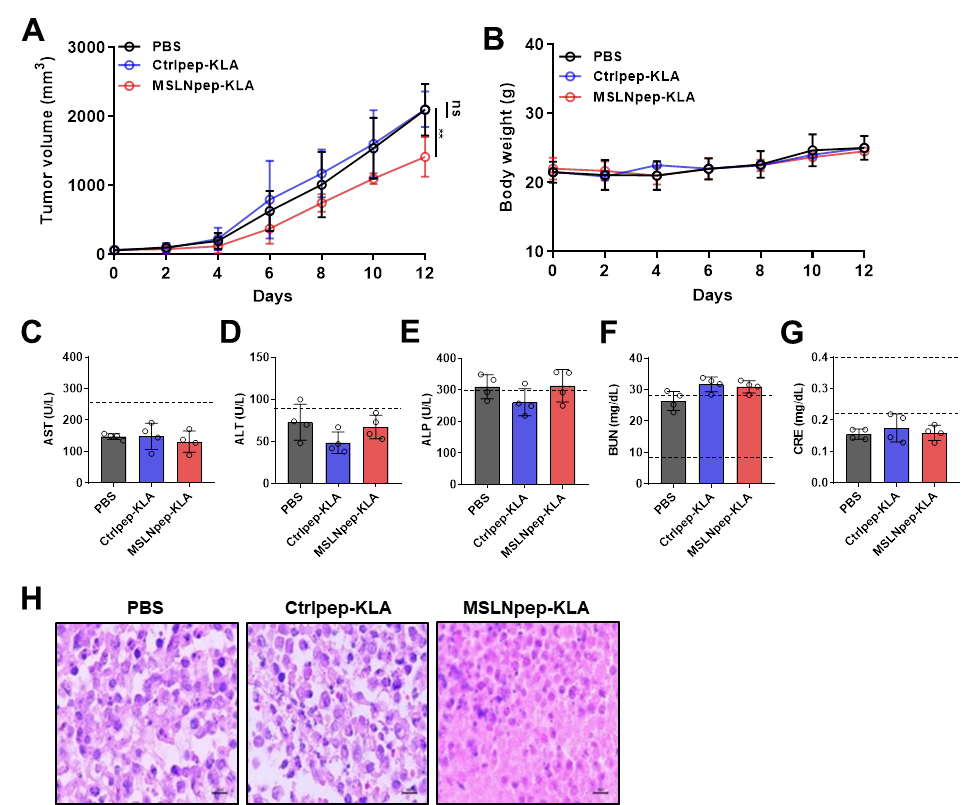


**Figure S10. Inhibition of subcutaneous pancreatic tumor growth in mice by MSLN–KLA. (A)** Mice bearing subcutaneous AsPC-1 pancreatic tumors were treated with phosphate-buffered saline (PBS), control peptide (Ctrlpep)–KLA, or MSLNpep–KLA (10 mg/kg body weight, i.v.). Tumor volumes were measured during treatment. **, *P* < 0.01; ns, significant by two-way ANOVA. **(B)** Body weights. (**C–G)** Serum levels of aspartate transferase (AST; C), alanine transferase (ALT; D), alkaline phosphatase (ALP; E), blood urea nitrogen (BUN; F), and creatinine (CRE; G) after treatment. **(H)** Hematoxylin and eosin staining of tumor tissues after treatment.

**
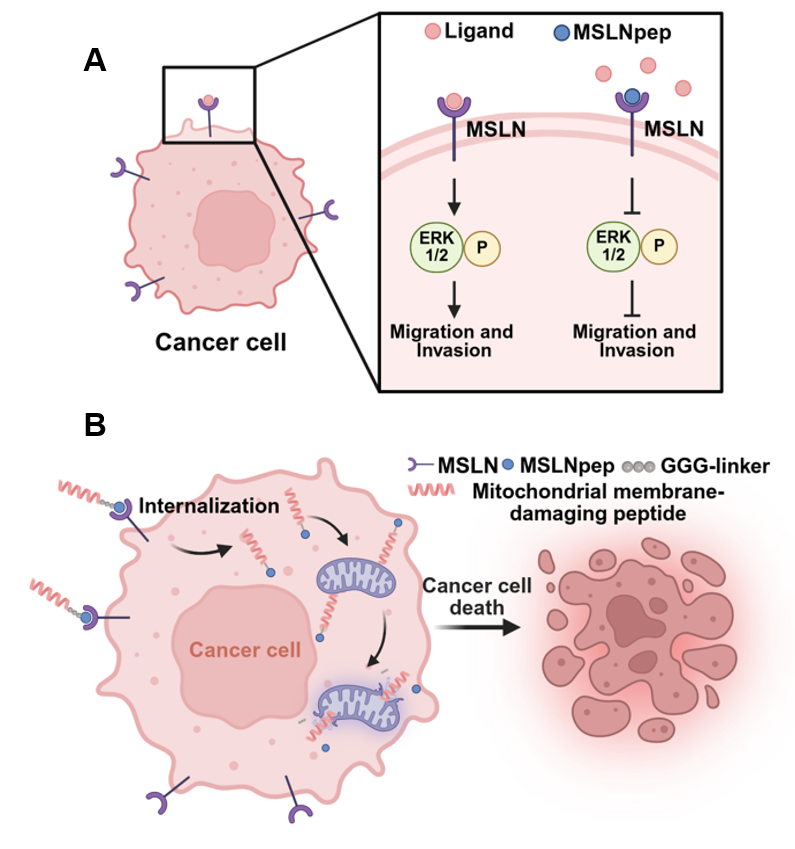
**

**Figure S11. Working models of MSLNpep and an MSLN-targeted mitochondrial membrane–damaging peptide. (A)** Binding of MSLN with its natural ligand induces phosphorylation of Erk1/2 and subsequent migration and invasion of MSLN-expressing tumor cells. MSLNpep binds MSLN and suppresses ligand-mediated downstream signaling, reducing cell migration and invasion. **(B)** The MSLN-targeted mitochondrial membrane–damaging peptide (a chimera of MSLNpep and a mitochondrial membrane–damaging peptide) binds MSLN and is internalized into MSLN-high tumor cells, where it disrupts the mitochondrial membrane and induces tumor cell death. Created with BioRender.com.
